# Optimizing Radiation Dose in High-Resolution Chest CT: The Impact of Patient-Specific Factors and Size-Specific Dose Estimates

**DOI:** 10.3390/diagnostics15060740

**Published:** 2025-03-16

**Authors:** Mohamed Abuzaid

**Affiliations:** 1 Medical Diagnostic Imaging Department, College of Health Science, University of Sharjah, Sharjah P.O. Box 27272, United Arab Emirates; mabdelfatah@sharjah.ac.ae; 2 Research Institute for Medical and Health Sciences, University of Sharjah, Sharjah P.O. Box 27272, United Arab Emirates

**Keywords:** HRCT, computed tomography, CT dose index, dose-length product, radiation dose, size-specific dose estimates

## Abstract

**Background/Objectives:** High-resolution chest computed tomography (HRCT) is a critical diagnostic tool, but radiation dose optimization remains a significant concern. Traditional dose metrics such as the volume CT dose index (CTDIvol) and dose-length product (DLP) do not adequately account for patient size variations. This study aimed to assess the radiation dose in HRCT using size-specific dose estimates (SSDEs) and evaluate the influence of patient-specific factors on key dosimetric parameters. **Methods**: This retrospective cohort study analyzed HRCT scans from 1970 adult patients conducted between September 2022 and February 2024. Radiation dose data, including the CTDIvol, DLP, SSDE, and effective dose, were extracted from the DoseWatch™ software. Patient demographics, scan protocols, and exposure parameters were collected. Descriptive statistics, correlation analyses, and significance testing were conducted using IBM SPSS (Version 26). **Results**: A significant positive correlation was found between the radiation dose parameters (CTDIvol, DLP, SSDE) and patient body size metrics, particularly BMI (rpb = 0.445, *p* < 0.01). The SSDE values ranged from 2.7 to 12.4 mGy, providing a more patient-specific dose assessment than traditional indices. Gender differences were observed, with male patients receiving higher radiation doses (*p* < 0.01). The scanning range exhibited a significant negative correlation with the CTDIvol and SSDE, suggesting dose variations with anatomical coverage. **Conclusions**: SSDEs provide a more accurate, patient-centered dose assessment in HRCT, allowing for optimized radiation safety strategies. These findings emphasize the need for size-adapted scan protocols to minimize exposure while maintaining diagnostic image quality. The routine integration of SSDE into clinical practice is recommended to enhance individualized dose management in HRCT.

## 1. Introduction

High-resolution chest computed tomography (HRCT) has become an essential diagnostic tool in evaluating thoracic pathologies due to its superior spatial resolution and ability to depict fine lung details. However, the high radiation dose associated with HRCT remains a critical concern, particularly because traditional dose metrics such as the volume CT dose index (CTDIvol) and dose-length product (DLP) do not account for variations in patient size [1,2]. The size-specific dose estimate [3,4,5] (SSDE) concept has emerged as a more patient-centered approach to quantifying the absorbed radiation dose to address these limitations. SSDEs adjust the CTDIvol based on patient dimensions, offering a more accurate reflection of the radiation dose received by the individual. Advancements in CT technology have played a pivotal role in reducing the radiation dose while preserving the diagnostic image quality. CT scanners now have automatic exposure control (AEC) systems that modulate the tube current in real time based on the patient attenuation measured during the initial scout scan. These systems ensure that radiation is delivered most efficiently, reducing unnecessary exposure while maintaining image quality [6,7]. Additionally, the ability to automatically adjust the tube voltage (kVp) according to the patient size and diagnostic requirements further contributes to dose efficiency.

Iterative reconstruction techniques and advances in detector technology enable the production of high-quality images from low-dose data by effectively reducing noise and contributing to significant dose reductions by improving the detector efficiency and spatial resolution [8,9].

A fundamental component in personalized radiation dose management is the implementation of SSDEs. Unlike conventional dose indices offering a one-size-fits-all approach, the SSDE provides a more individualized estimate of radiation exposure by incorporating patient-specific measurements, such as the effective or water-equivalent diameter. By applying a conversion factor to the CTDIvol, SSDEs adjust for differences in patient size, thereby offering a more realistic assessment of the absorbed dose [3,4,5]. This adjustment is particularly important when comparing doses across patients with varying body sizes. For example, a fixed CTDIvol may result in an unnecessarily high dose for a small adult or child.

In contrast, a larger patient might require a higher output simply due to increased tissue attenuation. The SSDE bridges this gap by normalizing the dose estimate, ensuring that the delivered radiation is appropriate for the patient’s morphology [10]. As such, the SSDE is an indispensable tool for both clinical dose monitoring and protocol optimization in HRCT.

Personalized dose management in HRCT tailors scan protocols by adjusting tube current, reducing the tube voltage, and limiting the scan range, which are key strategies. At the same time, high-pitch scanning further cuts the scan time and cumulative dose. This customization minimizes radiation exposure and enhances safety without compromising the diagnostic performance, marking a shift from uniform to more patient-centered protocols [2,11,12].

The current clinical guidelines and best practices strongly advocate for the incorporation of SSDEs into routine dose assessment and protocol optimization. Professional organizations such as the American Association of Physicists in Medicine (AAPM) and the American College of Radiology (ACR) have emphasized the importance of using patient-specific metrics when evaluating radiation doses [5,13]. These guidelines recommend that SSDEs be included in dose reports alongside traditional dose indices, facilitating more meaningful comparisons across different patient populations. In addition, establishing diagnostic reference levels (DRLs) that are stratified by patient size has been suggested as a practical means of benchmarking and improving radiation safety [14]. Such stratification allows facilities to identify and address instances where doses exceed those achievable for a given patient category. In conclusion, the evolution of personalized radiation dose management in HRCT represents a significant advancement in the pursuit of patient safety.

This study aims to assess the radiation dose in high-resolution chest CT (HRCT) by implementing size-specific dose estimates (SSDEs). By analyzing a retrospective cohort of 1970 patients, the study examines how patient-specific factors—such as body habitus, age, and clinical indication—influence key dosimetric parameters (DLP, CTDI_vol, SSDE, and effective dose).

## 2. Materials and Methods

### 2.1. Study Design and Data Collection

Patients were identified using the Picture Archiving and Communication System (PACS), which facilitated the selection of HRCT studies. Relevant clinical data, including patient demographics, examination protocols, and radiation exposure parameters, were extracted from the Radiology Information System (RIS) and cross-referenced with the patient’s medical records for completeness and accuracy.

This study was designed as an observational retrospective cohort analysis, evaluating radiation doses associated with HRCT examinations conducted between September 2022 and February 2024. A total of 1970 adult patient records were selected based on the availability of complete dose measurement data obtained through General Electric’s DoseWatch™ software. The study inclusion and exclusion criteria referred to HRCT scans lacking critical dosimetric parameters, including the CTDI_vol, DLP, SSDE, or patient demographics (age, gender, weight, height). These were excluded to ensure a robust analysis and maintain accuracy. Pediatric cases (patients younger than 18 years) and examinations utilizing intravenous contrast media were excluded due to inherent differences in scan protocols and radiation sensitivities, which could introduce variability.

Given the study’s retrospective nature, the institutional review board waived the requirement for informed consent. Ethical approval was obtained in compliance with institutional and national guidelines for research involving human data.

### 2.2. CT Equipment and Techniques

All data were acquired using an Optima CT660 CT scanner (GE Healthcare, Milwaukee, WI). The data were collected using the DoseWatch software by GE Healthcare (Milwaukee, Wis) for CT dose management. This software monitors, tracks, and analyzes the dose data and consequently suggests strategies for dose reduction. The patient’s HRCT chest scan was conducted without intravenous contrast media.

Patient positioning was strictly maintained in the supine orientation with the arms elevated above the head for all scans, verified by routine technologist training and periodic audits of adherence. The breath-hold duration was uniformly set at 7–8 s, and technologists consistently instructed patients to comply to reduce variability related to respiratory motion. Additionally, the CT scanner underwent routine daily calibration and monthly quality assurance checks as per institutional quality assurance standards to ensure consistent performance and accurate dosimetric measurements throughout the study.

Table 1 presents the scan protocol parameters, including the number of images per series and the scanning length, ranging from 302.1 mm to 499.6 mm, with a mean of 399.1 mm. This reflects the standard coverage for HRCT chest imaging. The exposure parameters during the scans included the tube voltage (kVp), tube current (mA), exposure time, and pitch factor. The mean kVp was 112.5, with a tube current averaging 162.1 mA. The exposure time per rotation was consistent at 0.5 s. These parameters highlight the standardized acquisition settings that ensure optimal image quality while maintaining radiation dose control. The CT scanner was disinfected after each scan as part of the routine clinical practice.

### 2.3. Effective Dose

The effective dose was calculated using the VirtualDose™ CT software from Virtual Phantoms, Inc. VirtualDose™ is a web-based tool that employs anatomically realistic phantoms representing patients of various ages and body types, from newborns to morbidly obese individuals. Built on a database derived from Monte Carlo simulations, the software integrates the ICRP-60 and ICRP-103 guidelines for effective dose estimation. Using the CTDIvol, DLP, and patient-specific data, the software provides accurate, effective dose calculations compatible with modern CT scanners [15,16].

### 2.4. Data Analysis

Data were analyzed in SPSS in IBM SPSS Version 26. Descriptive statistics of the gender, BMI groups, study protocol, and study description were calculated. Continuous variables were described as means ± standard deviation (SD) and medians and 25th and 75th percentiles. The correlation was evaluated using the point-biserial correlation and Pearson’s correlation coefficient. Results were considered statistically significant when the *p*-value < 0.01.

## 3. Results

### 3.1. Patients’ Demographics and Protocol Description

A total of 1970 adult patients were included, with nearly equal distribution between males (50.7%, *n* = 999) and females (49.3%, *n* = 971). The mean age was 51.9 years (range 18–96). Most patients were overweight (BMI 25–29.9; 48.0%, *n* = 946), representing the predominant subgroup relevant for dose optimization strategies, followed by those with a healthy weight (26.6%, *n* = 524), obesity (25.3%, *n* = 498), and underweight (0.1%, *n* = 2). Slightly over half of the scans (56.4%, *n* = 1112) were follow-up HRCT scans, underscoring the importance of radiation dose optimization in repeated examinations. Among the clinical indications, dyspnea was the most frequent (30.9%, *n* = 608), followed by pulmonary thromboembolism (25.2%, *n* = 497), pneumonia (24.3%, *n* = 478), dyspnea with expectoration (10.3%, *n* = 203), and consolidation (9.3%, *n* = 184) (Table 2). The distribution highlights frequent indications where personalized radiation dose management is particularly beneficial (Table 2).

### 3.2. Dosimetric Analysis of HRCT Chest Scans

Table 3 provides a detailed statistical analysis of the dosimetric parameters from the HRCT chest scans, including the DLP, CTDIvol, SSDE, and ED. The analysis encompasses the study population’s mean, standard deviation (SD), minimum and maximum values, and the 25th, 50th (median), and 75th percentiles.

The mean DLP was 161.14 mGy·cm, with a standard deviation of 80.92 mGy·cm, ranging from 48.9 to 486.9 mGy·cm. The distribution indicates that 25% of scans had DLP values below 83.8 mGy·cm, 50% were below 150.4 mGy·cm, and 75% were below 231 mGy·cm. For the CTDIvol, the mean was 4.01 mGy, with a standard deviation of 2.03 mGy, and the values ranged from 1.2 to 12.1 mGy. The 25th, 50th, and 75th percentiles for the CTDIvol were 2.0, 3.8, and 5.9 mGy, respectively, reflecting the variability in the scan protocols.

The SSDE, a metric tailored to the patient size, had a mean value of 5.56 mGy, with a standard deviation of 1.33 mGy, ranging from 2.7 to 12.4 mGy. The 25th, 50th, and 75th percentiles were 5.0, 5.6, and 6.0 mGy, respectively, indicating that most scans had SSDE values tightly clustered around the median. Finally, the effective dose (E103) averaged 3.24 mSv, with a standard deviation of 1.2 mSv, ranging from 2.3 to 4.9 mSv. The percentiles for the E103 were 3.09, 3.1, and 3.36 mSv, showing minimal variation in the effective dose distribution across the cohort.

**Figure 1:**
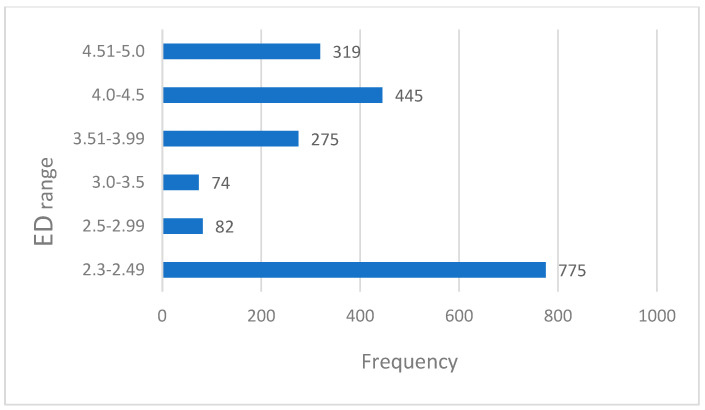
Effective dose frequency distributions.

Figure 1 shows the frequency distribution of the effective dose (ED) ranges in the HRCT scans. The most frequent range is 2.3–2.49 mSv (775 scans), followed by 4.0–4.5 mSv (445 scans) and 4.51–5.0 mSv (319 scans). Lower ranges, 2.5–2.99 mSv and 3.0–3.5 mSv, have the fewest scans (82 and 74, respectively). The distribution highlights the variability in the ED levels, with most scans in lower dose ranges, reflecting dose optimization practices and adherence to safety guidelines.

### 3.3. Correlation Analysis of Patient Factors and Radiation Dose Parameters

A point-biserial correlation was run to determine the relationship between the patient dose and gender. There was a positive correlation between the DLP and gender, which was statistically significant (rpb = 0.186, *n* = 1970, *p* = 0.000). Furthermore, there was also a positive correlation between the mean CTDIvol and gender, which was statistically significant (rpb = 0.135, *n* = 1970, *p* = 0.000). There was also a positive correlation between the SSDE and gender, which was statistically significant (rpb = 0.57, *n* = 1970, *p* = 0.11).

Pearson’s correlation was run to determine the significance of the relationship between the dose and BMI. There was a statistically significant positive correlation between the DLP and BMI (rpb = 0.445, *n* = 1970, *p* = 0.000), the mean CTDIvol and BMI (rpb = 0.307, *n* = 1970, *p* = 0.000), and the SSDE and BMI (rpb = 0.161, *n* = 1970, *p* = 0.000).

The Pearson correlation determined the relationship between the scanning range and patient dose. There was a negative correlation between the mean CTDIvol and the scanning range, which was statistically significant (rpb = −0.115, *n* = 1970, *p* = 0.000). Furthermore, a statistically significant negative correlation was found between the SSDE and scanning range (rpb = −0.92, *n* = 1970, *p* = 0.000). The Pearson’s correlation analysis conducted to study the correlation between the scanning range and image slices yielded a statistically significant positive correlation (rpb = 0.517, *n* = 1970, *p* = 0.000).

## 4. Discussion

This study investigated the radiation dose in HRCT chest scans, focusing on the SSDE and its influencing factors [3,5]. A total of 1970 adult patients were included in this retrospective analysis. The results indicate a statistically significant positive correlation between the DLP, CTDIvol, and SSDE and gender and BMI [10,17,18]. Specifically, male patients and those with higher BMI values tended to receive higher radiation doses. This finding aligns with previous research demonstrating the influence of the patient size on the radiation dose in CT [19]. Larger patients require higher mAs, increasing the radiation dose; gender differences reflect body size and composition [20]. Our results can be compared with those reported by Ghetti et al. (2020), who similarly investigated radiation dose parameters in HRCT scans, emphasizing patient-specific dose management. Both studies identified significant positive correlations between radiation dose indices (CTDI_vol, DLP, and SSDE) and patient body size parameters, particularly the BMI. Our mean CTDI_vol (4.01 mGy) and SSDE (5.56 mGy) values were somewhat lower compared to Ghetti et al. (6.8 mGy and 8.7 mGy, respectively) [21].

While previous studies have investigated patient-specific radiation dose management, the current study significantly expands on existing knowledge by employing a notably large retrospective cohort of 1970 adult patients, thus enhancing the robustness and generalizability of the results. Moreover, our application of the SSDE in clinical practice underscores its practical utility in real-world settings, moving beyond theoretical frameworks to demonstrate direct clinical applicability. This study also reveals previously unexplored correlations, particularly highlighting significant relationships between patient attributes such as BMI, gender, the scanning range, and radiation dose parameters (CTDIvol, DLP, SSDE). These findings underscore the necessity of personalized scan protocols, contributing practical insights for the refinement of dose optimization strategies and patient safety initiatives in clinical practice.

A negative correlation was observed between the CTDIvol and SSDE and the scanning range. This suggests that scans covering a larger anatomical region may result in a lower dose per section. This could be due to the automatic exposure control systems adjusting the tube current based on the total scan length, potentially lowering the dose in larger scans to maintain an acceptable overall radiation burden. However, the strong negative correlation between the SSDE and scanning range (rpb = −0.92) warrants further investigation to understand the underlying mechanisms and potential implications. The positive correlation between the scanning range and image slices is important, indicating that larger scan ranges naturally involve more slices. The positive correlation between the DLP and gender (rpb = 0.186) is statistically significant but relatively weak. This suggests that, while gender does influence the radiation dose, it may not be as strong a predictor as the BMI [20,22].

The finding that the indication has the strongest influence (41.5%) underscores the importance of tailoring scan protocols to the specific clinical indication. This suggests that a “one-size-fits-all” approach to CT protocols [23] is suboptimal and that dose optimization strategies should consider the diagnostic information sought.

As expected, the patient size, represented by the maximum patient thickness (19.1%) and maximum patient width (10.4%), plays a significant role in determining the radiation dose. This reinforces the need for patient-specific dose modulation, where the scan parameters are adjusted based on individual patient characteristics. Techniques like automatic exposure control and iterative reconstruction can help to optimize the image quality while minimizing the dose in larger patients.

The influence of age (12.2%) on the radiation dose is also noteworthy. This might reflect age-related variations in tissue sensitivity to radiation or differences in the scan protocols used for different age groups.

While the BMI, weight, and height contribute to the dose, their influence is less pronounced than the indication, patient thickness, and age [24,25]. This suggests that these factors, while relevant, may not be as critical for dose optimization as the primary drivers identified by the model.

Recent studies further support the clinical benefits of implementing SSDE-based personalized protocols in HRCT to optimize the radiation dose while maintaining diagnostic accuracy. Our results highlight the importance of size-adapted HRCT protocols, particularly for follow-up imaging, where cumulative radiation exposure is a concern. Studies have shown that applying SSDE-based dose adjustments enables significant dose reduction while preserving the diagnostic accuracy, particularly in lung screening and chronic disease monitoring. For instance, Saltybaeva et al. (2016) demonstrated that ultra-low-dose HRCT with size-specific adaptations reduced the lung cancer screening doses to sub-millisievert levels, minimizing the radiation risk while maintaining lesion detectability. Maintaining image quality and diagnostic accuracy is paramount when lowering the CT dose [26]. Recent studies indicate that SSDE-personalized protocols can reduce doses without compromising the diagnostic performance. May et al. (2021) implemented indication-specific HRCT scan protocols (tailored for fine fibrosis details, nodule detection, or pneumonia assessment). They found that more than half of the patients benefited from dose cuts using these individualized settings, with no loss in the interpretability of the findings [27].

### Limitation

This study’s retrospective design introduces potential selection bias due to the reliance on existing records, limiting control over variations in patient demographics and imaging parameters. The single-center data may also limit the generalizability to wider patient populations with differing protocols and patient profiles. Future multi-center studies are recommended to enhance the external validity and investigate the influence of advanced image reconstruction techniques on the dose and diagnostic image quality. Despite these limitations, this study provides valuable insights into the optimization of HRCT scan protocols to balance radiation safety and image quality.

## 5. Conclusions and Recommendations

This study evaluated radiation doses in high-resolution chest CT (HRCT), emphasizing the use of size-specific dose estimates (SSDEs) to improve patient-specific dose management. Significant positive correlations were found between dosimetric parameters (CTDIvol, DLP, SSDE) and patient-specific factors, especially the BMI and gender. Additionally, the negative correlation between the scanning range and SSDE highlights the importance of tailoring scan protocols according to the anatomical coverage. These findings highlight the importance of routinely integrating SSDEs into clinical practice through automated calculation and reporting in CT dose-monitoring software. Clinical protocols should be tailored explicitly by categorizing patients based on their BMI and clinical indication, with specific exposure parameters (e.g., tube current and voltage adjustments) set accordingly. Additionally, institutions should establish size-specific diagnostic reference levels (DRLs) and provide ongoing staff training to ensure protocol adherence and continuous improvement in radiation safety and diagnostic accuracy.

## Figures and Tables

**Table 1:** HRCT chest scan protocol and exposure parameters.

Parameter	Mean ± SD	Min–Max
Images per series	65.4 ± 7.4	44–93
Scanning length (mm)	399.1 ± 34.3	302.1–499.6
Exposure time (ms)	3263.1 ± 304.8	2019.9–7199.9
Tube voltage (kVp)	112.5 ± 10.1	100–140
Tube current (mA)	162.1 ± 53.2	72.2–300
Pitch factor	1.5 ± 0.0001	1.5–1.5
Exposure time per rotation (s/rot)	0.5 (constant)	0.5–0.5

Data from HRCT chest scan include patient information (age, gender, weight, height), examination data (study description, protocol name, series name, series description, number of scans), acquisition data (scan region, distance source to detector, generator power (kW), reconstruction diameter, distance source to the patient, focal spot, slice thickness, spacing between slices, convolution kernel, images per series, filter type, scan length), exposure parameters (KVP, tube current, pitch factor, exposure time per rotation, exposure time), and exposure data (size-specific dose estimates for body examinations (SSDE), volumetric computed tomography dose index (CTDIvol), dose-length product (DLP)).

**Table 2:** Patients and procedural characteristics: descriptive statistics.

		*n* (%)
Gender	Female	971 (49.28)
	Male	999 (50.71)
BMI Range	Underweight	2 (0.1)
	Healthy Weight	524 (26.59)
	Overweight	946 (48.02)
	Obesity	498 (25.27)
Study Protocol	First Time	858 (43.55)
	Thorax FU	1112 (56.44)
Indications	Consolidation	184 (9.34)
	Dyspnea	608 (30.86)
	Dyspnea and Expectoration	203 (10.3)
	Pneumonia	478 (24.26)
	Pulmonary Thromboembolism	497 (25.22)

**Table 3:** The mean values, the SD, and the 25th, 50th, and 75th percentiles for the DLP, CTDIvol, SSDE, and E103 reported for the total population.

			Percentile		
	Mean ± SD	Min–Max	25	50	75
DLP (mGy·cm)	161.14 ± 80.92	48.9–486.9	83.8	150.4	231
CTDIvol (mGy)	4.01 ± 2.03	1.2–12.1	2.0	3.8	5.9
SSDE (mGy)	5.56 ± 1.33	2.7–12.4	5.0	5.6	6.0
E103 (mSv)	3.24 ± 1.2	2.3–4.9	3.09	3.1	3.36

## Data Availability

Data will be available upon request to the corresponding author.

## Abbreviations

HRCT	High-Resolution Computed Tomography
CT	Computed Tomography
CTDIvol	Volume Computed Tomography Dose Index
DLP	Dose-Length Product
SSDE	Size-Specific Dose Estimate
AEC	Automatic Exposure Control
kVp	Automatic Exposure Control
DRLs	Diagnostic Reference Levels
AAPM	American Association of Physicists in Medicine
ACR	American College of Radiology
BMI	Body Mass Index
ICRP	International Commission on Radiological Protection
RIS	Radiology Information System
PACS	Picture Archiving and Communication System
ED	Effective Dose
SD	Standard Deviation
